# Is venous blood a more reliable description of acid-base state following simulated hypo- and hyperventilation?

**DOI:** 10.1186/s13049-021-00848-8

**Published:** 2021-02-17

**Authors:** Lisha Shastri, Søren Kjærgaard, Peter S. Thyrrestrup, Stephen E. Rees, Lars P. Thomsen

**Affiliations:** 1 Respiratory and Critical Care (RCare) group, Aalborg University, Aalborg, Denmark; 2 Department of Anaesthesiology and Intensive Care, Aalborg University Hospital North, Aalborg, Denmark

**Keywords:** Acid-base, Blood gas analysis, Arterial, Venous, Hyperventilation, Hypoventilation

## Abstract

**Background:**

ABGs are performed in acute conditions as the reference method for assessing the acid-base status of blood. Hyperventilation and breath-holding are common ventilatory changes that occur around the time of sampling, rapidly altering the ‘true’ status of the blood. This is particularly relevant in emergency medicine patients without permanent arterial catheters, where the pain and anxiety of arterial punctures can cause ventilatory changes. This study aimed to determine whether peripheral venous values could be a more reliable measure of blood gases following acute changes in ventilation.

**Methods:**

To allow for characterisation of ventilatory changes typical of acutely ill patients, but without the confounding influence of perfusion or metabolic disturbances, 30 patients scheduled for elective surgery were studied in a prospective observational study. Following anaesthesia, and before the start of the surgery, ventilator settings were altered to achieve a + 100% or − 60% change in alveolar ventilation (‘hyper-’ or ‘hypoventilation’), changes consistent with the anticipation of a painful arterial puncture commonly encountered in the emergency room. Blood samples were drawn simultaneously from indwelling arterial and peripheral venous catheters at baseline, and at 15, 30, 45, 60, 90 and 120 s following the ventilatory change. Comparisons between the timed arterial (or venous) samples were done using repeated-measures ANOVA, with post-hoc analysis using Bonferroni’s correction.

**Results:**

Arterial blood pH and PCO_2_ changed rapidly within the first 15–30s after both hyper- and hypoventilation, plateauing at around 60s (∆pH = ±0.036 and ∆PCO_2_ = ±0.64 kPa (4.7 mmHg), respectively), with peripheral venous values remaining relatively constant until 60s, and changing minimally thereafter. Mean arterial changes were significantly different at 30s (*P* < 0.001) when compared to baseline, in response to both hyper- and hypoventilation.

**Conclusion:**

This study has shown that substantial differences in arterial and peripheral venous acid-base status can be due to acute changes in ventilation, commonly seen in the ER over the 30s necessary to sample arterial blood. If changes are transient, peripheral venous blood may provide a more reliable description of acid-base status.

## Introduction

In emergency or intensive care medicine, blood gas analysis is used to monitor the acid-base and oxygenation status of the patient. Small disturbances around the time of sampling could induce acute transient changes in ventilation and misrepresentation of the acid-base status. Such transient changes could be due to anxiety or fear of the needle prior to puncture [1], resulting in hyperventilation or breath holding. Additionally, these changes could also be seen after alterations in ventilator settings or spontaneous breathing in ICU patients [2]. Although common practice is to wait 10 min or more to measure blood gases after a change in ventilator settings [3], it does not preclude the presence of acute transient changes around the time of sampling, thereby clouding clinical interpretation.

Transient factors affecting blood gases are poorly understood. Studies have demonstrated rapid arterial or venous changes in response to changes in ventilation, but in a non-standard way with volunteers simply requested to hyperventilate to a fixed respiratory frequency or hold their breath [4–6]. Short-term change in ventilation primarily affects blood pH and PCO_2_ with little effect on oxygenation unless inspired oxygen levels are low. Due to a larger volume and buffering capacity, the venous blood may not immediately match the changes observed in the arterial blood. This may have several implications related to the interpretation of blood gases at the bedside. Understanding the responses to acute transient changes in both arterial and venous blood are important concepts to consider while interpreting blood gases in emergency and intensive care.

This study investigates the acute response of end-tidal CO_2_ (EtCO_2_) and, simultaneous arterial and peripheral venous acid-base status, following standardised changes in ventilation, with responses to both an increase and decrease in ventilation being examined.

## Methods

### Patients

This was a prospective observational study where patients were studied in a surgical milieu allowing for standardisation of ventilator settings. Adult patients without cardiovascular or respiratory disease, scheduled for an elective robot-assisted laparoscopic prostatectomy or hysterectomy were included in the study. This study population allowed for the isolation and characterisation of ventilatory changes to acid-base status without the confounding influence of changes in perfusion, or the presence of metabolic disturbances, known to delay the transit of blood through the tissues. The study was conducted at Aalborg University Hospital North, Aalborg, Denmark, from August 2019 to March 2020, and written informed consent obtained from all the participants prior to the study. The study was approved by the Regional Ethical Committee for North Jutland and abides by the guidelines set in the Declaration of Helsinki.

### Protocol

The study procedure was performed after the patient was anaesthetised but prior to the start of the surgery, during the time when the patient is being prepared for surgery. Patients were anaesthetised according to the department protocol using total intravenous anaesthesia for the duration of the study, with arterial and peripheral venous catheters in-situ. The arterial catheter was inserted into *arteria radialis* and peripheral venous catheter into a vein in the *anterior cubital fossa*. Each patient was randomised to receive changes in mechanical ventilation corresponding to either ‘hyperventilation’ or ‘hypoventilation’ as described below.

#### Study procedure and blood sampling

Blood sample pairs were taken by two trained individuals, simultaneously drawing an arterial and a venous sample. The individuals were aware of the changes in ventilation as well as the catheter from which they were sampling, however, we do not expect this to result in systematic bias in relation to the sampling. Sample pairs were taken at baseline (2 pairs immediately after each other) and then at 15, 30, 45, 60, 90 and 120 s after the change in ventilation. Blood was collected in pre-heparinised *safe*PICO syringes (Radiometer, Copenhagen, Denmark) with 1 mL of blood per sample. The syringes were capped, and air bubbles removed immediately after sampling. The baseline samples were analysed prior to the start of the experiment, to allow all samples to be analysed within 30 min of sampling without storage on ice, as recommended by IFCC guidelines [7]. A third person ensured correct timing of the sampling and assisted in capping the syringes at the time of sampling. Samples were analysed in the order that they were taken, arterial before venous, on the same ABL800 Flex analyser (Radiometer, Copenhagen, Denmark).

#### Ventilator settings

Ventilator settings at baseline and for ‘hyperventilation’ and ‘hypoventilation’ are illustrated in Table 1. Patients were ventilated using IPPV ventilation with a volume-controlled mode (Dräger Primus^®^, Lübeck, Germany). Changes in ventilation approximated to either a 100% increase or 60% decrease in alveolar ventilation (V̇_A_). V̇_A_ was calculated from tidal volume (V_T_), anatomical dead space (V_D_) and respiratory rate (RR) from V̇_A_ = (V_T_ - V_D_) **∙** RR, assuming a V_D_ of 150 ml. For example, in a patient with an ideal body weight of 75 kg and baseline V_T_ = 6 ml/kg and RR = 15 breaths per min (bpm), V̇_A_ would equal ((6 **∙** 75) - 150) **∙** 15 = 4500 ml/min. A 100% increase, and 60% decrease, in V̇_A_ would result in values of 9000 and 1800 ml/min, respectively. Typically, in the emergency department (ED), hyperventilation can result in respiratory rates as high as 30 bpm and hypoventilation can present as complete apnea if the patient holds their breath. As complete cessation of ventilation was not desirable for this protocol, a reduction of 60% was considered safe by the study team. An increase of normal respiratory rate from 15 to 30 bpm represents a 100% increase in alveolar ventilation, we therefore selected an increase in 100%, however increasing both tidal volume and respiratory rate, so as to simulate a more normal pattern of breathing for the patient. EtCO_2_ and SpO_2_ were monitored throughout the study; EtCO_2_ continuously measured using Beacon Caresystem (Mermaid Care A/S, Nørresundby, Denmark) and SpO_2_ using pulse oximetry. The acute transient change in ventilation lasted 2 min so as to characterise typical short-term changes in ventilation.

**Table 1:** Ventilatory settings during baseline and for hyperventilation and hypoventilation

Parameters	Baseline	Hyperventilation	Hypoventilation
Tidal volume (V_T_)	6 ml/kg	8 ml/kg	6 ml/kg
Respiratory Rate (RR)	15 bpm	20 bpm	6 bpm
PEEP	5 cmH_2_O	5 cmH_2_O	5 cmH_2_O
Criteria for termination		EtCO_2_ < 1.5 kPa	sPO_2_ < 88% EtCO_2_ > 6.5 kPa

### Sample size calculation

The studies by Montagna et al. [5] and Steurer et al. [4] examined the effect of hyperventilation on blood gases. From their results we approximated the changes from baseline for PCO_2_ in both arterial and venous blood respectively, following voluntary hyperventilation. We used the mean and SD at baseline of 0 ± 0.5 kPa reported in [5], and the arteriovenous difference in PCO_2_ of 0.85 kPa, reported in [4, 5], 1 min into the hyperventilation period. For the SD at 1 min, we expected a slightly higher variance than that at baseline, and therefore chose an SD of 0.8 kPa. The calculated sample size using the data from baseline and at 1 min was therefore 12 patients each, for the hyper- and hypoventilation parts of the study, with α = 0.05 and β = 0.80. Ethical approval was obtained to study 30 patients.

### Statistical analysis

Variability of the arterial and venous blood were examined using the two pairs at baseline, which were taken immediately after each other. This was analysed using a Bland-Altman (BA) comparison, calculating biases and limits of agreement (LoA) for arterial and venous pH and PCO_2_ at baseline under steady state conditions for ventilation.

Changes following hyper- or hypoventilation were analysed as changes (∆) in pH, PCO_2_ and EtCO_2_ from baseline. Tests of normality were performed using Shapiro-Wilk’s test, and the data were found to be normally distributed. Comparisons between the timed arterial samples were analysed using a repeated measures ANOVA followed by a post-hoc analysis using Bonferroni’s correction to compare differences between the average values at each timepoint from baseline. Similar analyses were done for the peripheral venous samples and the EtCO_2_ measurements. All results are presented as mean ± SD, with *P* < 0.05 considered statistically significant. Statistical analysis was conducted on SPSS v25 (SPSS^©^ IBM Corp., Armonk, USA).

## Results

Thirty patients were studied, with 15 patients each in the hyper- and hypoventilation parts of the study. The mean age of the patients were 61 years and 27% were female. Further details are presented in Table 2.

**Table 2:** Population characteristics (*n* = 30), baseline blood gas values are a mean of two samples taken immediately after each other

Parameters	Mean ± SD or N (%)
Age (years)	61 ± 10
Sex- Female	8 (26.7)
Height (cm)	176 ± 7
Weight (kg)	84.8 ± 12.6
Surgery	
Prostatectomy	22 (73.3)
Hysterectomy	8 (26.7)
Arterial (baseline)	
pH	7.409 ± 0.035
PCO_2_ (kPa)	5.55 ± 0.64
PCO_2_ (mmHg)	41.63 ± 4.80
Venous (baseline)	
pH	7.391 ± 0.034
PCO_2_ (kPa)	6.02 ± 0.73
PCO_2_ (mmHg)	45.15 ± 5.48
EtCO_2_ (baseline; kPa)	4.67 ± 0.62
EtCO_2_ (baseline; mmHg)	35.03 ± 4.65

### Variability of pH and PCO_2_ at baseline steady-state ventilation

**Fig. 1:**
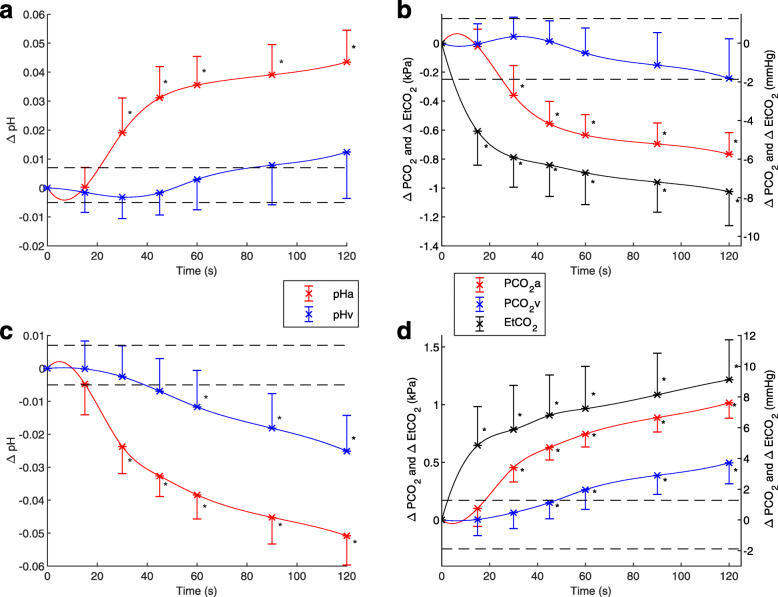
Changes in end-tidal CO_2_, arterial and peripheral venous pH and PCO_2_ (kPa, mmHg) in response to hyper- (**a, b**) and hypoventilation (**c, d**). Changes in end-tidal CO_2_ (EtCO_2_; black), arterial (red) and peripheral venous (blue) pH and PCO_2_ plotted as a change from baseline (∆) in response to hyperventilation (**a** & **b**) and hypoventilation (**c** & **d**). Data presented as mean ± SD. Error bars (only one side shown) represent SD at that time point. Dotted black horizontal lines represent the widest LoAs of the variability at baseline in the arterial and peripheral venous blood for pH (**a** & **c**) and PCO_2_ (**b** & **d**) in the respective graphs. *statistically significant with *P* < 0.05 when compared to baseline using a repeated measures ANOVA followed by a post-hoc test with Bonferroni’s correction

The bias and LoA of pH and PCO_2_ values at baseline ventilation were, for arterial blood 0.001 (− 0.004 to 0.006) and − 0.11 (− 0.25 to 0.03) kPa (or − 0.83 (− 1.88 to 0.22) mmHg), and for peripheral venous blood 0.001 (− 0.005 to 0.007) and − 0.01 (− 0.20 to 0.17) kPa (or 0.08 (− 1.50 to 1.28) mmHg), respectively (BA plots not shown). The widest LoAs of the variability of the arterial and venous pH and PCO_2_ are incorporated in Fig. 1 as horizontal dotted lines to represent the blood’s inherent variability.

### Responses to hyper- and hypoventilation

The changes from baseline (∆) in EtCO_2_, arterial and peripheral venous blood, are shown in Fig. 1 as averages and SD (one-sided) for all patients. Changes in pH, PCO_2_ and EtCO_2_ in response to hyperventilation are displayed in Fig. 1a & b and in response to hypoventilation in Fig. 1c & d, respectively. No patient reached termination criteria during the course of the study.

Following an acute transient increase in ventilation, arterial pH and PCO_2_ changed faster than in peripheral venous blood, with a rapid and statistically significant change from baseline initiated within the first 30s (*P* < 0.001), plateauing around 60s (∆pH = 0.036 ± 0.010 and ∆PCO_2_ = − 0.64 ± 0.14 kPa (− 4.77 ± 1.07 mmHg)). Venous blood parameters changed after 90s, remaining minimal and not significantly different from baseline. An acute transient decrease in ventilation changed arterial pH and PCO_2_ faster than hyperventilation, with changes in arterial acid-base status observed as early as 15s. Arterial values of pH and PCO_2_ were significantly different from baseline at 30s (*P* < 0.001), with responses reaching − 0.038 ± 0.007 and 0.75 ± 0.11 kPa (5.59 ± 0.85 mmHg) for ∆pH and ∆PCO_2_, respectively, at 60s. Venous pH was significantly different from baseline at 60s (*P* < 0.01) and PCO_2_ at 45s (*P* < 0.002). Arterial PCO_2_ followed the trend of EtCO_2_ with a minimal delay of 15s between them in response to both hyper- and hypoventilation. EtCO_2_ was significantly different from baseline 15s after the change in ventilation (*P* < 0.001).

## Discussion

Acid-base and oxygenation status are important measurements, needed for the management of patients in intensive and emergency care. Acute transient changes in ventilation can rapidly affect these parameters in arterial blood. This study has shown that standardised increases and decreases in ventilation result in a reproducible pattern of rapid changes in arterial pH and PCO_2_ seen as quickly as 30s in accordance with the speed of changes in the lungs (EtCO_2_). Peripheral venous blood is less responsive to these changes, presenting first after 60s, meaning venous blood essentially filters out changes due to short term variability in ventilation. Previous studies have shown that voluntary hyperventilation can induce a rapid change in the arterial (5) or venous (13) blood, with a maximal change observed at ~ 2 min for arterial and ~ 4 min for venous blood. The lack of standardisation in these studies make it difficult to determine and compare the precise response of blood gases to the changes in ventilation, with this study being the first to investigate responses to standard changes in ventilation.

Repeated blood sampling is associated with anxiety and fear in the patient, especially in those with chronic illnesses [8]. This anxiety could manifest due to numerous reasons, for example in anticipation of the next puncture or in response to the pain of the needle prick [8–10], thereby precipitating momentary changes in respiratory patterns including hyperventilation and breath-holding [11, 12], commonly encountered in the emergency room, in admitted patients with COPD, or for awake patients on non-invasive ventilation. Transient changes can also be observed in intensive care, when patients on assisted ventilation engage in spontaneous breathing [2]. Janssens et al. [13] studied the response of hypercapnic patients to initiation or interruption of non-invasive positive pressure ventilation and demonstrated a change in PaCO_2_ of 2–2.7 kPa (15–20 mmHg), within the first 2 min, which are much larger than the changes evidenced in this study.

In a survey conducted by Rang et al. [14], discussing the clinical significance of variation in blood gases, emergency physicians suggest that a difference in PCO_2_ of 0.8 kPa (6 mmHg) is clinically significant. In our study, we see an average change in PaCO_2_ of 0.64 and 0.75 kPa at the end of 1 min in response to hyperventilation and hypoventilation, which in combination with the SDs describing variation at baseline gives a range of ~ 1 kPa (7.5 mmHg), at or above the clinically significant range described by Rang et al. The transient changes in arterial acid-base seen here many be similar to, or even lower than, those seen in departments of emergency or pulmonary medicine, with the ventilatory changes applied in this study relatively small when compared to hyperventilation (up to 30 bpm) or breath-holding, frequently encountered in the ED. In addition, the duration of this protocol is consistent with brief periods of hyperventilation or breath holding and corresponds to the duration of a typical arterial puncture which takes around 30s to perform by a trained individual [9], with additional time required for patient preparation [15].

In contrast, peripheral venous blood showed a smaller and a more delayed response of over 30s longer than that in the arterial blood. A reason for this could be slowing down of blood flow in the peripheral capillaries in combination with the sluggish flow in the veins. The large volume of interstitial fluid that equilibrates with the arterial blood before it reaches the veins, could induce an additional, but minor delay between these two compartments [16–18]. While the significant differences between the arterial and venous systems described in this study are a result of isolated effects of ventilation, in the ED and other departments treating acutely ill patients, it is likely for there to be other problems, including poor perfusion and other metabolic issues. This will presumably further slow the transit of blood in the tissues, thereby further delaying the venous response.

Given the acute transient differences in acid-base status shown here, the question arises as to whether the venous or arterial gases are a more accurate, or perhaps stable, description of patient state. This study shows that if ventilation changes are acute and transitory, then the venous values might be a more reliable representation of the patient’s acid-base status, filtering out these transient changes, and providing further evidence for the increasing trend of using venous blood as a screening tool in the ED. If, however, changes are acute but non-transitory, i.e. maintained over a period of more than the couple of minutes investigated here, then it is clear that care should be taken to measure arterial samples in steady state, and to allow further time for the equilibration of venous blood, which is likely to be delayed in relation to arterial blood.

The major limitation of the study was that blood sampling was ended at 2 min to investigate the fast response due to acute transient changes in ventilation, and does not investigate the delay in venous blood in relation to arterial over a longer duration. Characterisation of these short-term dynamics, however, is novel, and no previous data exists with paired arterial-venous sampling performed every 15s.

## Conclusion

In conclusion, this study has shown that substantial differences in arterial and peripheral venous acid-base status can be due to acute changes in ventilation. If these changes are transient, peripheral venous blood may provide a more reliable description of acid-base status. Most importantly, if an ABG is used for quantification of acid-base status, these results indicate that it must only be in situations of stable ventilation.

## Data Availability

The datasets used and/or analysed during the current study are available from the corresponding author upon reasonable request.

## Abbreviations

ABG: Arterial blood gas
ANOVA: Analysis of variation
BA plot: Bland-Altman plot
COPD: Chronic obstructive pulmonary disease
EtCO_2_: End tidal carbon dioxide
ICU: Intensive Care Unit
LoA: Limits of agreement
PCO_2_: Partial pressure of carbon dioxide
SD: Standard deviation
SpO_2_: Peripheral saturation of oxygen
